# BCLAF1-induced HIF-1*α* accumulation under normoxia enhances PD-L1 treatment resistances via BCLAF1-CUL3 complex

**DOI:** 10.1007/s00262-023-03563-8

**Published:** 2023-10-31

**Authors:** Bowen Yao, Ye Lu, Yazhao Li, Yixue Bai, Xinyu Wei, Yuanyuan Yang, Demao Yao

**Affiliations:** 1 Department of Hepatobiliary Surgery, The First Affiliated Hospital of Xi’an Jiaotong University, Xi’an, China; 2 Department of Geriatric Surgery, The First Affiliated Hospital of Xi’an Jiaotong University, Xi’an, China; 3 Center for Translational Medicine, The First Affiliated Hospital of Xi’an Jiaotong University, Xi’an, China; 4 Xi’an Jiaotong University Health Science Center, Xi’an, China

**Keywords:** Atezolizumab, BCLAF1, Hepatocellular carcinoma, HIF-1*α*, Immunotherapy, PD-L1

## Abstract

Bcl-2-associated transcription factor-1 (BCLAF1), an apoptosis-regulating protein of paramount significance, orchestrates the progression of various malignancies. This study reveals increased BCLAF1 expression in hepatocellular carcinoma (HCC) patients, in whom elevated BCLAF1 levels are linked to escalated tumor grades and diminished survival rates. Moreover, novel BCLAF1 expression is particularly increased in HCC patients who were not sensitive to the combined treatment of atezolizumab and bevacizumab, but not in patients who had tumors that responded to the combined regimen. Notably, overexpression of BCLAF1 increases HCC cell proliferation in vitro and in vivo, while the conditioned medium derived from cells overexpressing BCLAF1 strikingly enhances the tube-formation capacity of human umbilical vein endothelial cells. Furthermore, compelling evidence demonstrates that BCLAF1 attenuates the expression of prolyl hydroxylase domain protein 2 (PHD2) and governs the stability of hypoxia-inducible factor-1*α* (HIF-1*α*) under normoxic conditions without exerting any influence on transcription, as determined by Western blot and RT‒qPCR analyses. Subsequently, employing coimmunoprecipitation and immunofluorescence, we validated the reciprocal interaction between BCLAF1 and Cullin 3 (CUL3), through which BCLAF1 actively upregulates the ubiquitination and degradation of PHD2. The Western blot and RT‒qPCR results suggests that programmed death ligand-1 (PD-L1) is one of the downstream responders to HIF-1*α* in HCC. Thus, we reveal the pivotal role of BCLAF1 in promoting PD-L1 transcription and, through binding to CUL3, in promoting the accumulation of HIF-1*α* under normoxic conditions, thereby facilitating the ubiquitination and degradation of PHD2.

**Supplementary Information:**

The online version contains supplementary material available at 10.1007/s00262-023-03563-8.

## Introduction

Hepatocellular carcinoma (HCC) is an important malignancy in the current medical landscape [1], affecting approximately 1,000,000 lives annually [2]. While surgical resection and liver transplantation are potential avenues for treatment, the foremost approach to combat advanced HCC lies in the administration of tyrosine kinase inhibitors (TKIs), such as sorafenib and lenvatinib [3–5]. Over the past few decades, a multitude of molecules have been implicated in the recurrence and metastasis of HCC [6]. However, the underlying molecular mechanisms through which HCC recurrence and metastasis occur continue to elude our comprehensive understanding. Among potential mechanisms, ferroptosis [7], the EGF–EGFR pathway [8], the hypoxic microenvironment [9], and matrix stiffness [10] have emerged as intriguing prospects for investigation. Therefore, uncovering novel factors involved in HCC holds implications for the advancement of therapies and design of pharmaceutical interventions.

Programmed death ligand-1 (PD-L1) represents a compelling and alluring therapeutic target in the realm of aggressive malignancies and has become the focus of attention from both researchers and clinicians. Its predominant presence has been observed in inflamed epithelial tissues, cancer cells, and host stromal cells. Thus, the expression of PD-L1 has been identified as a pivotal and prognostic biomarker that influences the response to anti-PD-1/PD-L1 treatments. Intriguingly, various clinical studies have shown that patients with heightened PD-L1 + host cells tend to derive greater benefits from anti-PD-1/PD-L1 therapy than do those with an abundance of PD-L1 + cancer cells [11]. Nevertheless, the precise nature, regulatory mechanisms, and functional attributes of HCC cells exhibiting elevated PD-L1 expression remain unclear and require further exploration and elucidation.

Bcl-2-associated transcription factor-1 (BCLAF1) is a pivotal regulator of apoptosis that engages in dynamic protein interactions with the adenoviral Bcl-2 homolog E1B19K [12]. The Bcl-2 family comprises both pro-apoptotic (Bad, Bax, or Bid) and anti-apoptotic (Bcl-2 and Bcl-XL) members, and the balance between these proteins dictates the responsiveness to apoptotic stimuli [13]. BCLAF1 has been implicated in a multitude of functions, including apoptosis, cell cycle progression, DNA repair [14, 15], and AML tumorigenesis [16]. A recent groundbreaking study showed that the translocation of BCLAF1 to the nucleus, which was facilitated by the upregulation of p53 and Bax expression, culminated in ischemia‒reperfusion injury [17]. Additionally, in HCC, ginsenoside compound K orchestrates BCLAF1 activation in the context of HIF-1*α*-mediated glycolysis [18]. Furthermore, BCLAF1 directly interacts with HIF-1*α*, thereby initiating transcription to augment HIF-1*α*-mediated angiogenesis under hypoxic conditions [19]. Nevertheless, we postulate that unexplored molecular mechanisms underlie the accumulation of HIF-1*α*.

Hypoxia-inducible factor-1 (HIF-1) exerts regulatory control over gene expression to adapt to varying oxygen concentrations. As elucidated in previous studies, the prolyl hydroxylase domain (PHD) family, von Hippel‒Lindau (VHL) protein, and proteasome collectively assume pivotal roles in the degradation of HIF-1*α* [20]. Under normoxic conditions, HIF-1*α* undergoes rapid degradation via the ubiquitin‒proteasome system, while HIF-1β remains stable in both aerobic and hypoxic cellular environments [21]. Thus, we postulate that the stability of HIF-1*α* is directly related to its activation [22]. Within well-oxygenated cells, PHD catalyzes the hydroxylation of HIF-1*α* using O_2_ and *α*-ketoglutarate (*α*KG), after which VHL associates with HIF-1*α*, triggering ubiquitination and subsequent proteasomal degradation [23].

This study unveils novel insights into HIF-1*α* accumulation under normoxic conditions. Consequently, we hypothesize that elevated levels of BCLAF1 govern the expression of PHD2, thereby facilitating the degradation of HIF-1*α*. This investigation was undertaken to substantiate the significance of BCLAF1 in this process and to elucidate the molecular mechanism through which HIF-1*α* accumulates in HCC. The results of this work hold promise for potential applications in HCC therapy and opens avenues for further research in this domain.

## Materials and methods

### Cell culture and transfection

Hep3B, MHCC97H, HepG2, 293T, and Huh7 cell lines were procured from the Cell Bank of the Chinese Academy of Sciences, located in Shanghai, China. HUVECs were procured from the American Type Culture Collection. Cells were grown in nutrient-rich DMEM (Gibco and Sigma) and supplemented with 10% FBS (Gibco, a division of Thermo Fisher Scientific). The growth medium was further fortified with a 1% solution of penicillin and streptomycin (Gibco). Cells were treated with CHX at a concentration of 40 μg/ml for 0, 3, 6, 8, and 12 h (+ Mg132 20 μM), before being harvested for subsequent analysis. The cells were transfected with the BCLAF1 overexpression plasmid, containing HG19747-CF (Sino Biology). Additionally, the cells were transfected with the CUL3 overexpression plasmid (NM_003590.4) (Gene Copeia). Transfections were performed using Lipo8000 (Beyotime), to ensure the transfer of genetic material with precision.

### Immunohistochemical staining

Twenty-five human hepatocellular carcinoma (HCC) tissue specimens were obtained from the First Affiliated Hospital of Xi’an Jiaotong University, Xian, China. For immunohistochemical analysis, the anti-BCLAF1 antibody (sc-101388, Santa) was used as previously described [24].

### Western blot analysis

The total proteome was extracted using RIPA lysis buffer (Beyotime), and protein concentration was ascertained utilizing a BCA kit. Next, 20 μg of protein was subjected to electrophoresis on 10% SDS‒PAGE gel, and the separated proteins were transferred onto 0.22 μm PVDF membranes (Roche, China). Then, the membrane was blocked with 5% nonfat milk for a duration of 2 h at ambient temperature. Afterward, the membrane was incubated with primary antibodies, including anti-BCLAF1 (sc-101388, Santa), anti-CUL3 (sc-166110, Santa), anti-HIF-1*α* (ab179483, Abcam), anti-PHD2 (ab133630, Abcam), anti-GAPDH (10,494–1-AP, Proteintech), and anti-Ubi (ab134953, Abcam), overnight at 4 °C. After washing with TBST, the PVDF membranes were incubated with secondary antibodies for 1 h. Thereafter, bound proteins were visualized after treatment with a chemiluminescence detection reagent (Millipore, USA).

### IP and coimmunoprecipitation assay

The proteins were extracted for Western blotting and immunoprecipitation (Beyotime) using cell lysis buffer. Next, protein G was incubated with 4 μg of primary antibodies (anti-BCLAF1 (sc-101388, Santa) or 4 μg of IgG for a duration of 30 min at room temperature. Afterward, the total protein pool was incubated with magnetic beads overnight at 4 °C. Elution buffer was employed to successfully isolate the proteins of interest, which were then detected using established Western blotting protocols. The gel was also stained with Coomassie blue for approximately 20 min before being destained overnight with the use of a destaining solution (Solarbio). For coimmunoprecipitation (Co-IP), the Pierce Classic Magnetic IP/Co-IP Kit (Thermo Fisher Scientific, USA) was used in accordance with the manufacturer instructions.

### Real‐time quantitative PCR (RT‐qPCR)

We isolated total RNA using TRIzol reagent and cDNA was synthesized using the RevertAid First Strand cDNA Synthesis protocol (K1622-Thermo Fermentas). Subsequently, mRNA levels were evaluated utilizing the SYBR Green PCR kit (Thermo Fisher Scientific) with CFX96 Touch Real-Time PCR (Bio-Rad). The following primers were employed for the amplification process:

BCLAF1: 5′-TCTGGAATAGAAGGCACTCTAGG-3′ and 5′-ACCCTCGTCTTTTAGAAACAGGA-3′;

PHD2: 5′-CCCAACGGGCAGACGAAGCC-3′ and 5′- CTTCCCGGTGTCGTGCAGGG-3′;

HIF-1*α*: 5′-CCCATTCCTCACCCATCAAATA-3′ and 5′- CTTCTGGCTCATATCCCATCAA-3′;

CUL3: 5′-TCAACCTCAACTCCAGGTCTCC-3′ and 5′-TGTTGCCTGAATTCATCCATCG-3′;

PD-L1: 5′-AAATGGAACCTGGCGAAAGC-3′ and 5′-GATGAGCCCCTCAGGCATTT-3′; and 

β-actin: 5′-ACTCGTCATACTCCTGCT-3′ and 5′-GAAACTACCTTCAACTCC-3′.

The experimental procedures were performed by employing cutting-edge technology and adhering to well-established protocols to ensure precise measurements.

### Cell Counting Kit-8 (CCK-8) assay

Hepatocellular carcinoma (HCC) cell lines, including Hep3B and Huh7, were seeded in a 96-well microplate. Subsequently, 10% CCK-8 solution (Enogene) was introduced at 24, 48, and 72 h. The cells were incubated for 2 h at 37 °C and quantified via optical density at 450 nm.

### Tube-formation assay

We placed recovered Matrigel solution (BD Biosciences, USA) into a 24-well plate and incubated the plate for 30 min. The established HUVECs (8 × 10^4^), having previously gown in the supernatant of BCLAF1-OE cells and BCLAF1-NC cells, were then placed onto the solidified Matrigel. The plates were incubated for 4–6 h at 37 ℃, visualized at 100 × magnification and quantified using ImageJ software.

### Immunofluorescence

Huh7 and Hep3B cells were cultured on confocal dishes for 24 h. For double immunofluorescence staining, cells were fixed with 4% paraformaldehyde and permeabilized with 0.2% Triton X‐100 and then incubated overnight at 4 °C with CUL3 (sc-166110)/BCLAF1 (sc-101388) and PHD2 (ab226890) antibodies. Then, the cells were incubated with anti‐rabbit‐conjugated antibodies and anti‐mouse‐conjugated antibodies at room temperature for 30 min in the dark. Finally, the nucleus was counterstained with DAPI.

### Statistical analysis

Statistical analysis was performed with GraphPad Prism 8.0.2, a powerful statistical software package. The data are presented as the mean ± standard error of the mean (SEM) obtained from three distinct experimental replicates. To evaluate the significant differences among the groups, Student’s *t* test and ANOVA were employed, which are established methods in scientific research. Noteworthy levels of significance are indicated as follows: *p < 0.05; **p < 0.01; and ***p < 0.001. These stringent criteria allow for accurate interpretation of the statistical outcomes.

## Results

### BCLAF1 was upregulated in patients with tumor progression after anti-PD-L1 treatments and was associated with HCC cell proliferation and angiogenesis

We obtained pathological data from patients suffering from HCC, a malignancy of the liver, which was deemed inoperable and was thus treated with a cutting-edge frontline immunotherapy protocol at our medical institution. Over the course of one year, a retrospective analysis was conducted on 32 patients who received the combined treatment of atezolizumab and bevacizumab, antibodies targeting PD-L1 and VEGF*α*, respectively, in the T + A regimen. Among these patients, eight cases comprising four cases of neoadjuvant treatment and four cases of conversion therapy exhibited remarkable success in both therapies. These groundbreaking outcomes open new avenues for potential repeat surgical resections or the implementation of safer resection procedures.

Given the intrinsic limitations associated with relying exclusively on histopathology for the diagnosis of liver cancer, a cohort of six patients with advanced hepatocellular carcinoma (HCC) presenting with portal vein tumor thrombosis (PVTT) were observed to exhibit disease progression after treatment, with only one case yielding diagnostic tissue upon biopsy. Consequently, our investigation centered on a subset of five patients with comprehensive pathological tissue sections. Notably, it is crucial to emphasize that among individuals experiencing treatment-related progression under the T + A therapeutic regimen, all exhibited concurrent PVTT. Drawing upon our team’s earlier explorations into the formation of PVTT, we postulate that key proteins significantly upregulated in liver cancer, and potentially implicated in PVTT genesis, may play pivotal roles in the progression of HCC following monoclonal antibody therapy targeting PD-L1 and VEGF*α*.

**Fig. 1:**
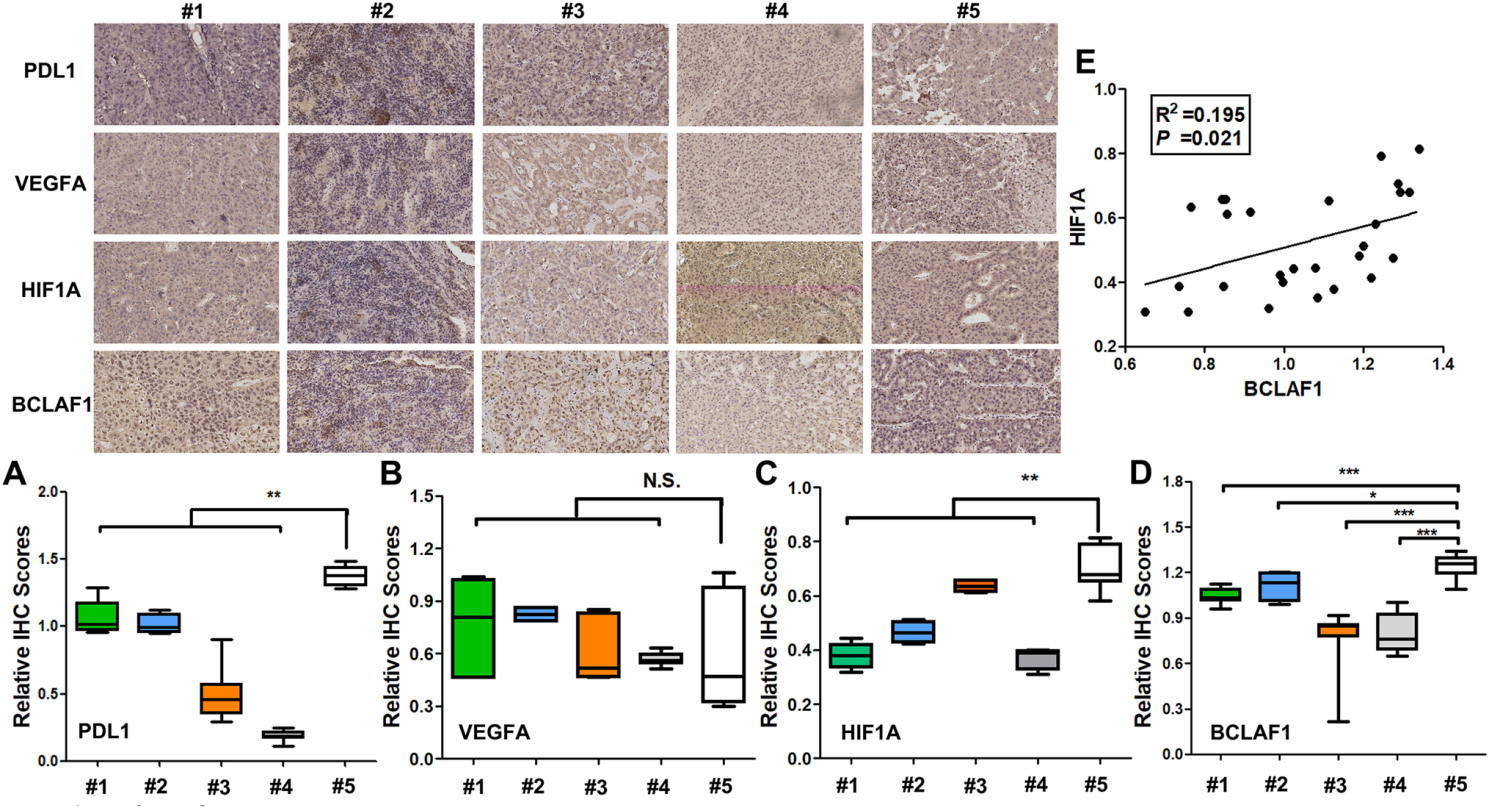
Protein expression profiles in hepatocellular carcinoma (HCC) samples treated with T + A therapy. Samples 1–4 were acquired from patients who exhibited favorable responses to the T + A regimen following surgical interventions. Sample 5 originated from a patient who experienced bone metastasis subsequent to T + A therapy **(A–D)**. Immunohistochemical (IHC) scores were determined for PD-L1, VEGF*α*, HIF1A, and BCLAF1 in all five samples **(E)**. a positive correlation was observed between BCLAF1 and HIF1A IHC scores across the analyzed samples

Initially, employing immunohistochemical (IHC) staining, we confirmed that among the four HCC samples from patients that effectively responded to treatment, the initial expression of PD-L1 was markedly higher than that in cases demonstrating disease progression following treatment. This observation aligns with similar findings in other malignancies, where some researchers hypothesize that elevated PD-L1 levels are indicative of treatment efficacy. Yet, this matter remains contentious in the context of liver cancer. Interestingly, no significant differences were observed in the initial expression of vascular endothelial growth factor A (VEGF*α*). Employing proteomic analysis of PVTT and immunostaining in fifteen pairs of PVTT samples, we identified key proteins implicated in PVTT, including cofilin 1 (CFL1), vasodilator-stimulated phosphoprotein (VASP), Bcl-2-associated transcription factor 1 (BCLAF1), hypoxia-inducible factor-1-alpha (HIF-1*α*), and certain members of the collagen family (which were markedly overexpressed in PVTT). Through IHC staining, we noted striking upregulation of BCLAF1 in samples demonstrating disease progression following T + A treatment (Fig. 1). Similarly, the expression level of HIF-1*α* was consistently higher in the treatment-progression group. Samples from the TA-sensitive group (#1–4) exhibited lower BCLAF1, HIF1*α*, and PD-L1 expression, while the expression of these proteins was higher in sample 5 HCC with bone metastasis after T + A treatment), as assessed by IHC staining. Furthermore, the level of BCLAF1 positively correlated with the level of HIF1*α* (Fig. 1E).

Although BCLAF1 has been extensively investigated in liver cancer, we identify a novel and intriguing role for this protein in liver cancer immunotherapy. The limited number of cases in our study prevents us from definitively establishing the correlation between BCLAF1 and the efficacy of immunotherapy. However, leveraging invaluable pathological data, our preliminary findings suggest that this relationship exists. These results may support the predictive capacity of BCLAF1 in evaluating the effectiveness of PD-L1 antibody therapy. To further unravel the intricate connection between BCLAF1 and immunotherapy, we performed subsequent experimental investigations.

**Fig. 2:**
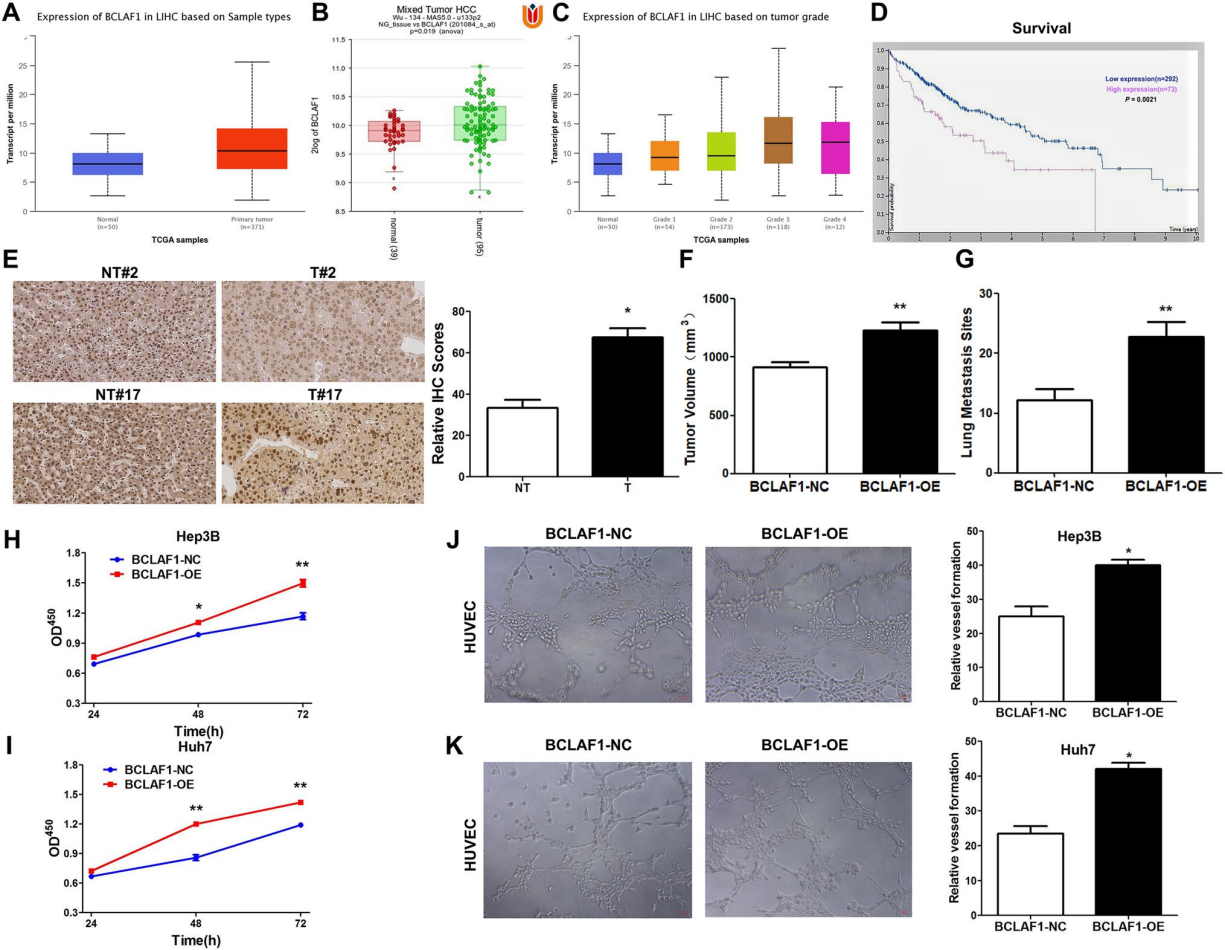
Expression and functionality of BCLAF1 in hepatocellular carcinoma (HCC) patients. (**A**, **B**). BCLAF1 expression in HCC patients was assessed using the comprehensive TCGA and GEO databases (**C**). An association was observed between tumor grades and the level of BCLAF1 expression in HCC patients (**D**). The Human Protein Atlas (HPA) provided an overall survival curve based on BCLAF1 protein expression in a cohort of 365 HCC patients (**E**). Immunohistochemistry analysis revealed higher BCLAF1 levels in 25 human HCC samples compared to their paired normal tissues (**F**, **G**). Overexpression of BCLAF1 resulted in increased tumor volumes and enhanced lung metastasis in mice (**H**, **I**) CCK-8 assays validated the significant enhancement of cell proliferation in HCC cell lines following transfection with BCLAF1 plasmids (**J**, **K**). BCLAF1 positively regulated the capacity of tube-formation in HUVEC cells. Specifically, HUVEC cells were seeded on Matrigel-coated wells at a consistent density of 8 × 10^4^ cells per well and incubated at 37 ℃ for 24 h before the addition of culture supernatant obtained from HCC cells transfected with BCLAF1 plasmids. Images were captured after 6–8 h (200 × magnification) and quantified using the ImageJ software. The presented results represent the mean ± SEMs of more than three independent experiments. Statistical significance was denoted as *p < 0.05 and **p < 0.01

Prior to our in vitro assays, we first extracted HCC data from The Cancer Genome Atlas (TCGA) and Gene Expression Omnibus (GEO) to explore the expression of BCLAF1. Notably, BCLAF1 expression was remarkably reduced in normal human liver tissues (Fig. 2A), which was corroborated by our GEO findings (Fig. 2B). Furthermore, a progressive increase in BCLAF1 expression with increasing HCC pathological grade became evident (Fig. 2C). Intriguingly, high levels of BCLAF1 emerged as a predictor of diminished overall survival in patients with HCC (Fig. 2D and Supplementary Fig. 1A). Moreover, through immunohistochemical staining, we confirmed that BCLAF1 expression was elevated in 25 cases of human HCC compared to their paired adjacent normal samples (Fig. 2E). After testing BCLAF1 expression in HCC cell lines, we chose Huh7 and Hep3B cells for further experiments (Supplementary Fig. 1B). In vivo, the overexpression of BCLAF1 accelerated tumor growth and lung metastasis in mice (Fig. 2F, G). These compelling findings strongly indicate the potential involvement of BCLAF1 in HCC progression, prompting us to conduct in vitro assays. The CCK-8 assay results unequivocally demonstrated that transfection with BCLAF1 overexpression plasmids enhanced the proliferation of Hep3B and Huh7 HCC cell lines (Fig. 2H, I). Additionally, given the well-established ability of human umbilical vein endothelial cells (HUVECs) to form capillary-like structures on Matrigel, we further ascertained that the culture supernatant from BCLAF1-overexpressing HCC cells significantly augmented the capacity of HUVECs to form more mature structures compared to the control groups (Fig. 2J, K). In summary, these compelling results underscore the potential of BCLAF1 expression as an underlying prognostic biomarker and reveal its pivotal role in the growth of HCC.

### BCLAF1 promotes HIF-1*α* expression under normoxic conditions

To determine the regulatory mechanism governing BCLAF1 expression in patients receiving immunotherapy, we next sought to explore the hypoxic tumor microenvironment, which interacts with the immune microenvironment. In examining the interplay between BCLAF1 and the hypoxic microenvironment, we obtained previously unreported results. Our prior dataset positioned BCLAF1 as a crucial transcription factor in the hypoxia-responsive gene network, controlling the expression of an array of genes. However, these discoveries seemingly diverged from the prevailing scientific discourse.

Previous research has indicated that BCLAF1 regulates HIF-1*α* transcription, even under oxygen-saturated conditions of normoxia. However, our focus on the hypoxic tumor microenvironment in liver cancer has led us to believe that the influence of BCLAF1 extends beyond the regulation of HIF-1*α* transcription during normoxic states. It has been demonstrated that under normoxic conditions, the PHD2-VHL pathway swiftly orchestrates the degradation of HIF-1*α*. However, our findings show that the overexpression of BCLAF1 during normoxia leads to the remarkable accumulation of HIF-1*α*. Therefore, BCLAF1 contributes to HIF-1*α* accumulation during normoxia through a potent inhibitory effect on HIF-1*α* degradation.

**Fig. 3:**
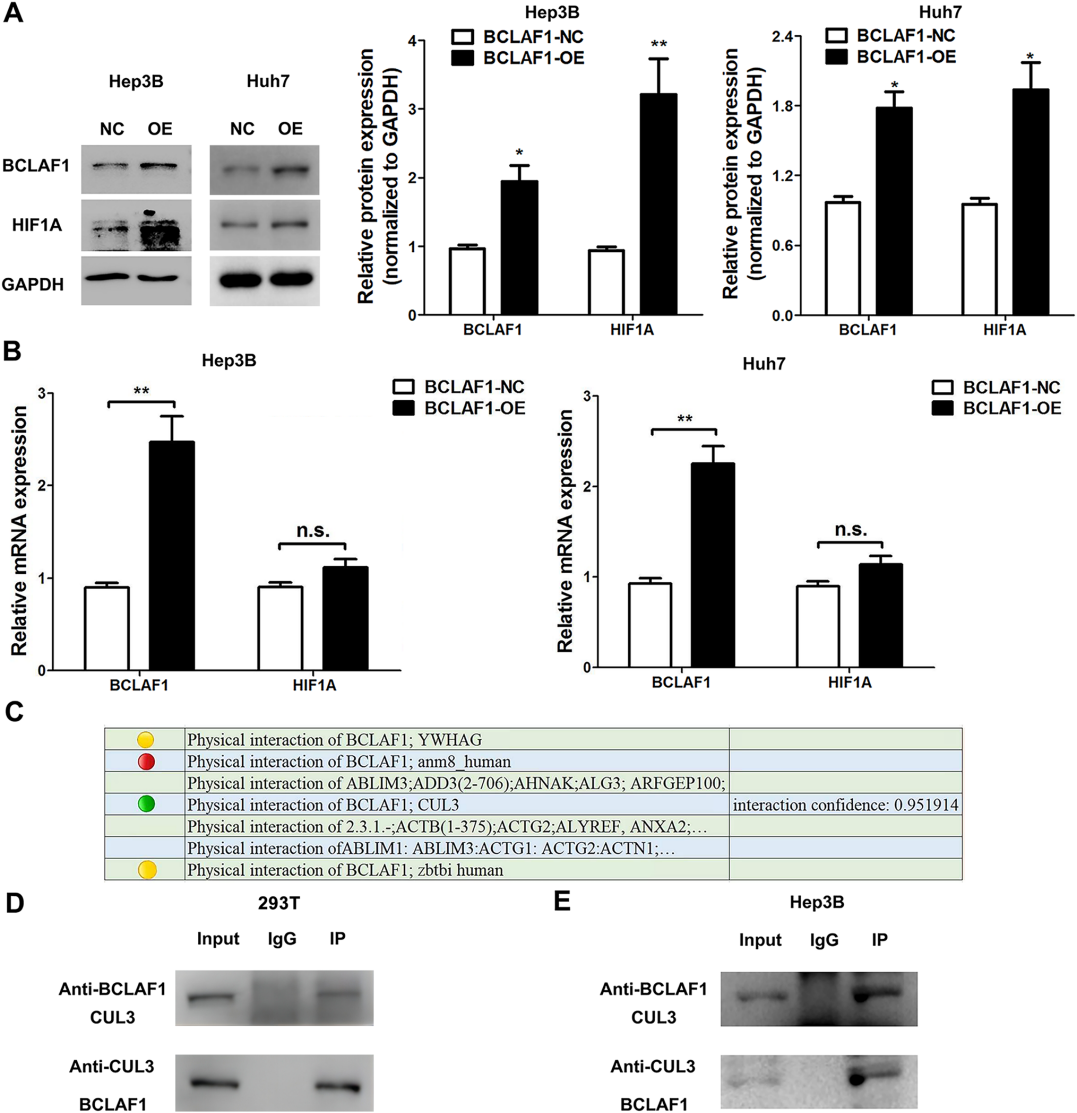
The expression of BCLAF1 exhibited a significant correlation with HIF-1*α* within hepatocellular carcinoma (HCC) cells (**A**). The levels of HIF-1*α* and BCLAF1 proteins were assessed in HCC cells subjected to transfection with an overexpression plasmid, along with the control group (**B**). The quantification of messenger RNA (mRNA) was accomplished through reverse transcription-polymerase chain reaction (RT-PCR) analysis (**C**).The physical interaction between BCLAF1 and CUL3 was ascertained based on data retrieved from the BioGRID database (**D**, **E**). The interaction between BCLAF1 and CUL3 was confirmed through coimmunoprecipitation (CO-IP) assays testing by 293 T and Hep3B cell lines. Statistical significance was denoted as *p < 0.05 and **p < 0.01

Previous inquiries have conclusively shown that BCLAF1 exerts an effect on the transcriptional activity of HIF-1*α* in 1% O_2_. To understand this phenomenon in greater detail, we transfected Hep3B and Huh7 cell lines with overexpression plasmids harboring BCLAF1 alongside the requisite control plasmids. Subsequent Western blot analysis revealed a distinct surge in HIF-1*α* protein levels in cells overexpressing BCLAF1 when compared with the control groups (Fig. 3A). Intriguingly, in contrast to prior findings, examination of mRNA levels revealed no alteration in HIF-1*α* mRNA levels (Fig. 3B). This seeming incongruity led us to postulate that BCLAF1 might modulate the stability of HIF-1*α* during normoxia. As established, HIF-1*α* undergoes hydroxylation by PHD2, interaction with the von Hippel Lindau (VHL) E3 ubiquitin ligase complex and proteasomal degradation [23, 25–27].

Through scrutiny of the literature and examination of databases [28–30], we unveiled the intrinsic mechanism by which CUL3 orchestrates PHD2 degradation. The CUL3-KEAP1 complex engages with the independent PHD2 protein, initiating a cascade of events that leads to the upregulation of polyubiquitination, ultimately causing PHD2 degradation. Notably, the interaction between BCLAF1 and CUL3 has come to the forefront.

Subsequently, examination of the BioGRID database revealed that BCLAF1 and CUL3 physically interact, revealing a molecular relationship between these proteins (Fig. 3C). The coimmunoprecipitation assay results indicate that the interaction between BCLAF1 and CUL3 is reciprocal (Fig. 3 D, E and Supplementary Fig. 1C). These findings collectively indicate that HIF-1*α* stability, under normoxic conditions, is controlled by BCLAF1.

### BCLAF1 stabilizes HIF-1*α* by promoting the ubiquitination and degradation of PHD2

**Fig. 4:**
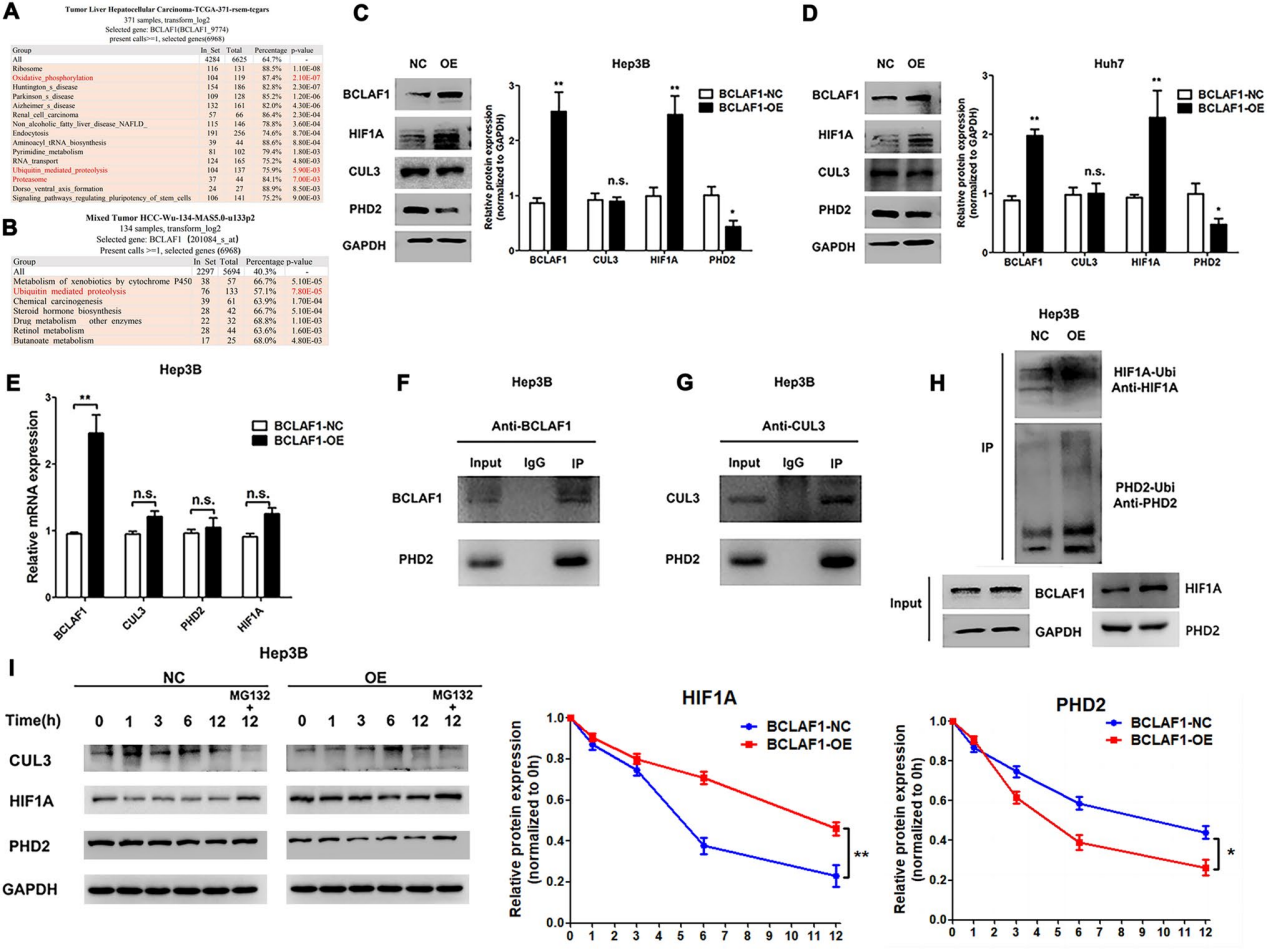
Regulation of PHD2 ubiquitination by BCLAF1 (**A**). KEGG enrichment analysis from TCGA showed that oxidative phosphorylation, ubiquitin-mediated proteolysis, and proteasome were enriched (**B**). Data from the GEO database showed ubiquitin-mediated proteolysis was enriched (**C**, **D**). BCLAF1 reduced the protein level of PHD2 and increased HIF-1*α*, but it did not affect CUL3 in Hep3B and Huh7 cells (**E**). RT-PCR showed there were no changes between the BCLAF1-overexpression and the control group (**F**, **G**). IP results showed that PHD2 combines with both BCLAF1 and CUL3 (**H**). IP-Ubi confirmed that BCLAF1 increased PHD2 ubiquitination and reduced the HIF-1*α* ubiquitination level (**I**). A protein degradation assay by CHX and Mg132 indicated that BCLAF1 promoted the degradation of PHD2 by the proteasome and resulted in the accumulation of HIF-1*α* protein. Statistical significance was denoted as *p < 0.05 and **p < 0.01. N.S. no significance

Upon confirming the interaction between BCLAF1 and CUL3, we extracted relevant data from the KEGG database, encompassing both TCGA and GEO databases. This comprehensive KEGG enrichment analysis revealed enhancement of ubiquitin-mediated proteolysis and the proteasome pathway (Fig. 4A, B).

The Western blot results revealed intriguing dynamics within the BCLAF1 overexpression group. Specifically, PHD2 levels were notably reduced, while HIF-1*α* levels were remarkably augmented. Notably, these alterations exerted no discernible influence on the CUL3 protein (Fig. 4C, D). Similarly, mRNA measurements revealed no significant variation in the expression of the aforementioned genes (Fig. 4E).

The findings from previous Co-IP experiments validated our initial hypothesis, supporting the existence of an interaction between BCLAF1 and CUL3. Armed with this insight, we performed a comprehensive investigation into the potential regulatory role of the BCLAF1-CUL3 complex in PHD2 expression. Co-IP assays provided further clarity, demonstrating the intricate association of PHD2 with CUL3 and BCLAF1 (Fig. 4F, G). Moreover, supplemental immunofluorescence experiments were performed to observe the colocalization of Bclaf1/CUL3 and PHD2 (Supplementary Fig. 2).

To gain deeper insights into the modulation of PHD2 ubiquitination by BCLAF1, we conducted immunoprecipitation and Western blot analyses. These experiments revealed a significant increase in PHD2 ubiquitination levels and a concomitant rise in HIF-1*α* levels following BCLAF1 overexpression (Fig. 4H).

Subsequently, a protein degradation assay was employed to elucidate the intricate dynamics at play. This assay showed that BCLAF1 overexpression resulted in the enhanced stabilization of HIF-1*α* and reduced stability of PHD2. Remarkably, the CHX chase assay results highlighted the propensity of BCLAF1 to increase the rate of PHD2 protein degradation while concurrently attenuating the degradation of the HIF-1*α* protein. However, CUL3 protein level remained unaffected.

CUL3-KEAP1 complex plays the role in protein degradation and we tested whether KEAP1 promotes the degradation of PHD2. Further results of Western blotting showed that upregulated BCLAF1 could not increase the protein level of CUL3 and KEAP1. However, decreased KEAP1 diminished PHD2 expression (Supplementary Fig. 3). These effects remained unswayed by the steadfast CUL3 protein. Notably, when protein synthesis was restrained, proteasome activation, as signified by MG132, mitigated the degradation of PHD2 in Hep3B cells (Fig. 4I).

In conclusion, the BCLAF1-CUL3 complex is shown to regulate PHD2 stability by orchestrating ubiquitination and protein degradation via the proteasome machinery.

### BCLAF1-CUL3 stabilizes HIF-1*α* and promotes HCC cell progression

**Fig. 5:**
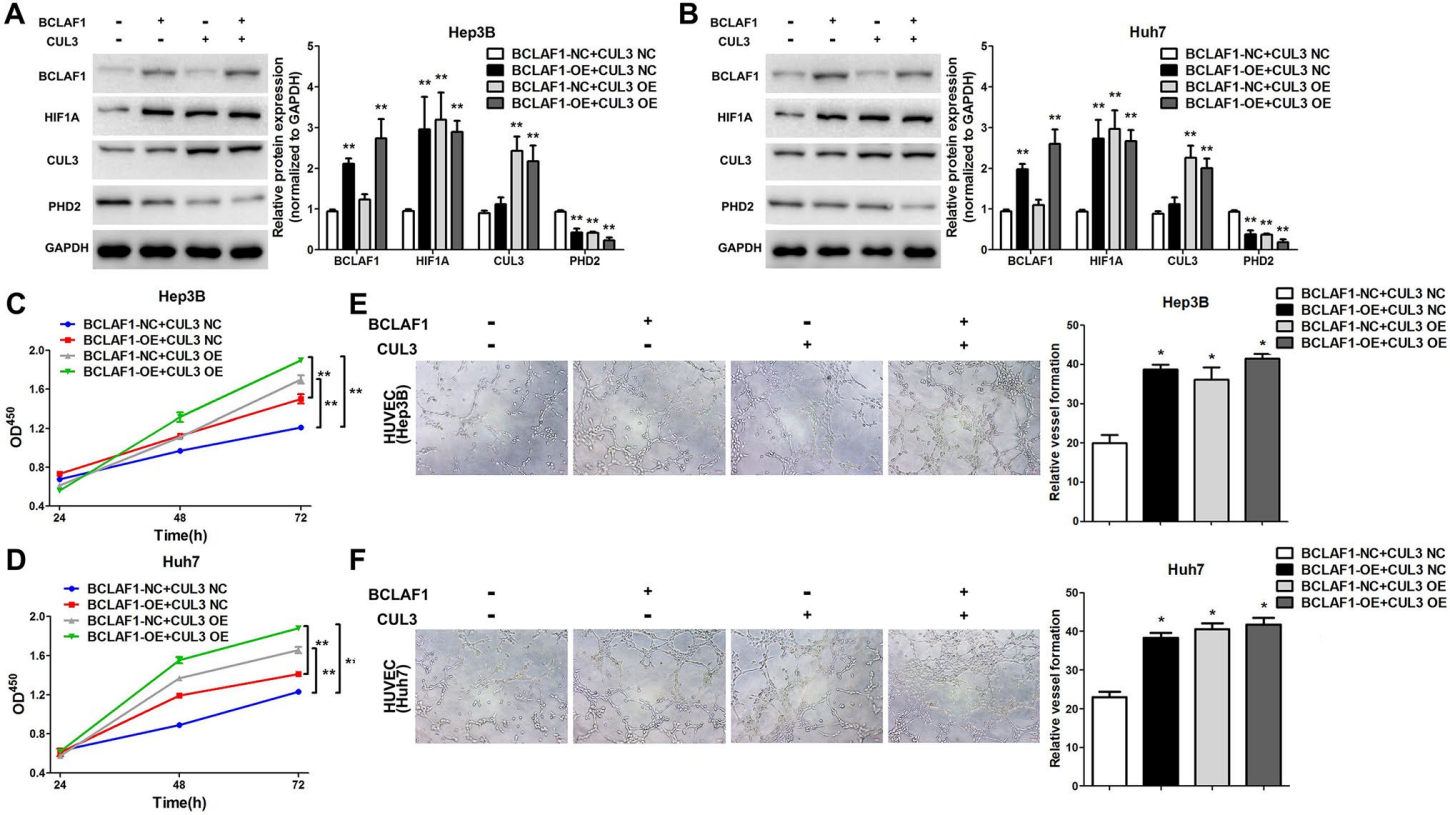
BCLAF1-CUL3 orchestrates the modulation of HIF-1*α* accumulation through the regulatory control of PHD2 (**A**, **B**). Cells were transfected with BCLAF1 or CUL3 overexpression plasmids individually or in combination. Notably, employing Western blot analysis, a remarkable reduction in PHD2 expression levels was observed, accompanied by a concomitant increase in HIF-1*α* expression (**C**, **D**). In order to evaluate the impact of selective BCLAF1 or CUL3 overexpression, as well as the synergistic effect of both, on cellular functions, we conducted CCK-8 cell proliferation assays and tube-formation assays using Hep3B and Huh7 cells (**E**, **F**). To offer a comprehensive understanding of the regulatory mechanism underlying BCLAF1-CUL3-mediated degradation of PHD2 and subsequent modulation of HIF-1*α* levels, we have provided a schematic illustration. Statistical significance was denoted as *p < 0.05 and **p < 0.01

To comprehend the implications of BCLAF1-CUL3-mediated degradation of PHD2, we endeavored to determine whether this intricate complex could influence the stability of PHD2, thereby modulating the abundance of HIF-1*α*. Given the pivotal role of PHD2 in suppressing the accumulation of HIF-1*α*, we hypothesized that the BCLAF1-CUL3 complex might exert its influence on HIF-1*α* levels by impacting PHD2. Our empirical evidence unequivocally revealed that overexpression of BCLAF1, CUL3, or both resulted in elevated levels of HIF-1*α* and concomitant decreases in PHD2 abundance while the CUL3 level remained unaltered (Fig. 5A, B). To substantiate these observations, CCK-8 assays confirmed that HCC cell lines transfected with BCLAF1 or CUL3 overexpression plasmids, independently or in combination, exhibited enhanced proliferative capacity (Fig. 5C, D). Moreover, our tube-formation assay findings demonstrated that the dual expression of BCLAF1 and CUL3 augmented the tube-forming potential of HUVECs (Fig. 5E, F). In summary, the BCLAF1-CUL3-mediated degradation of PHD2 leads to elevated levels of HIF-1*α* in HCC cells; however, other mechanisms may also be involved in the regulation of HIF-1*α*.

### Overexpression of BCLAF1 promotes PD-L1 transcription

Our preliminary findings suggest that BCLAF1 plays a pivotal role in facilitating the accumulation of HIF-1*α* under normoxic conditions, a phenomenon that has been tentatively substantiated in our clinical specimens (Fig. 1). In patients subjected to T + A therapy, while the presence of HIF-1*α* was discernible, its expression levels remained modest, and nuclear HIF-1*α* expression was infrequently observed. Nonetheless, a persistent positive correlation was observed between HIF-1*α* and PD-L1 expression. Whether the augmented levels of HIF-1*α* following increased BCLAF1 expression under normoxic conditions bear significant biological implications and contribute to alterations in PD-L1 expression is worthy of further investigation.

**Fig. 6:**
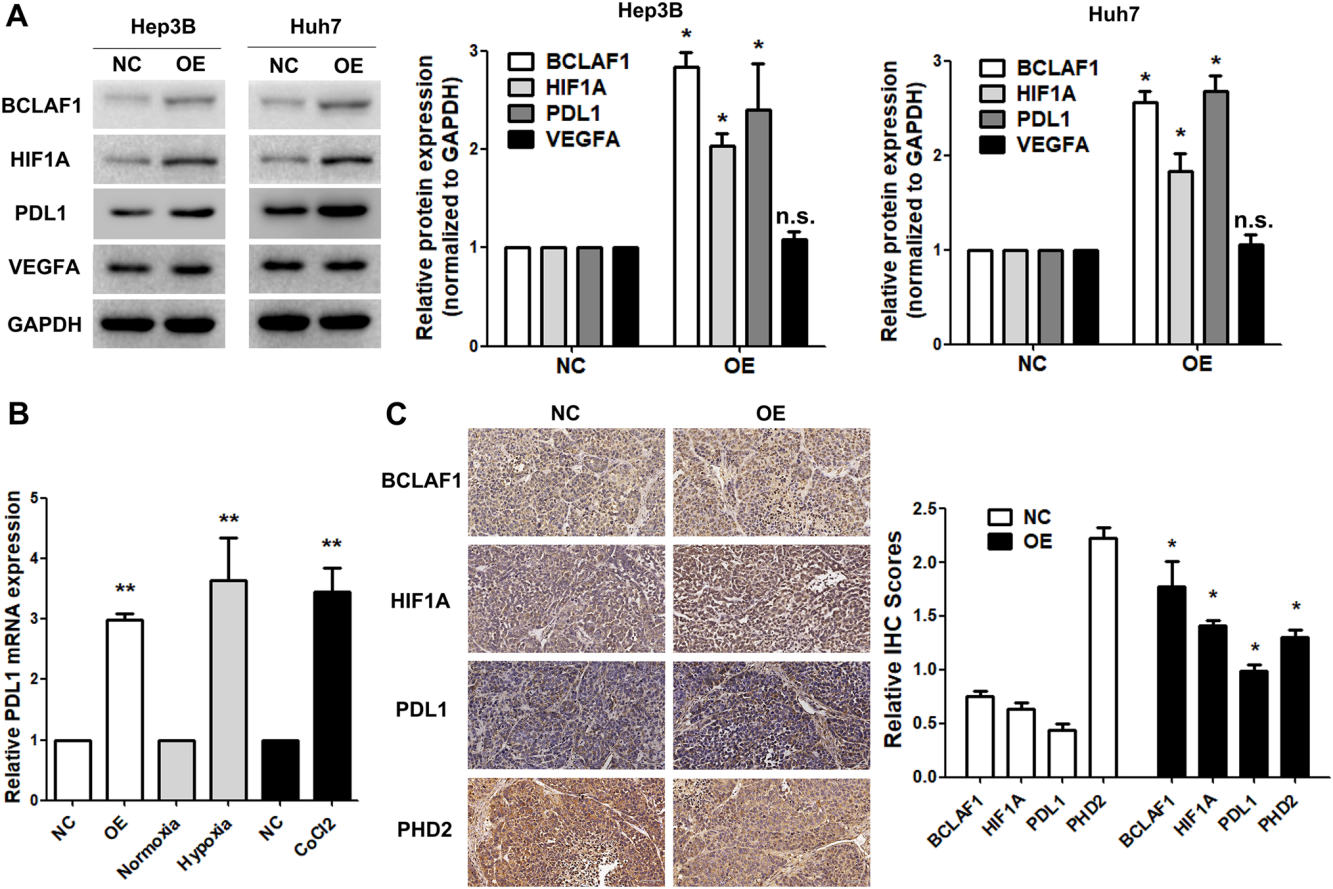
BCLAF1 promoted transcription of PD-L1 (**A**)Western Blot showed that the level of PD-L1 increased and HIF-1*α* increased, with no significant change of VEGF*α* via normoxia (**B**). qPCR results showed upregulated BCLAF1,under normoxia or physical or chemical stimuli of hypoxia could increase PD-L1 transcription (**C**) IHC staining of BCLAF1, HIF-1*α*, PD-L1 and PHD2 of HCC tumor nodules. Statistical significance was denoted as *p < 0.05 and **p < 0.01. N.S. no significance

In our original cellular models, the overexpression of BCLAF1 led to a notable increase in HIF-1*α* expression, which was paralleled by a corresponding increase in the protein levels of PD-L1 (Fig. 6A). Under normoxic conditions, as well as in the presence of chemical stimuli such as CoCl_2_ (a hypoxia inducer) and in hypoxic culture, the overexpression of BCLAF1 resulted in an upregulation of PD-L1 mRNA levels (Fig. 6B).

In vivo, we examined the levels of BCLAF1, HIF-1*α*, PD-L1, and PHD2 proteins in tumors. Significant differences in protein expression were observed, with higher levels of BCLAF1, HIF-1*α*, and PD-L1 detected in the OE group, while lower levels of PHD2 were observed in the same group (Fig. 6C). In summary, our findings suggest that PD-L1 is a responder that functions downstream of HIF-1*α* in HCC.

## Discussion

**Fig. 7:**
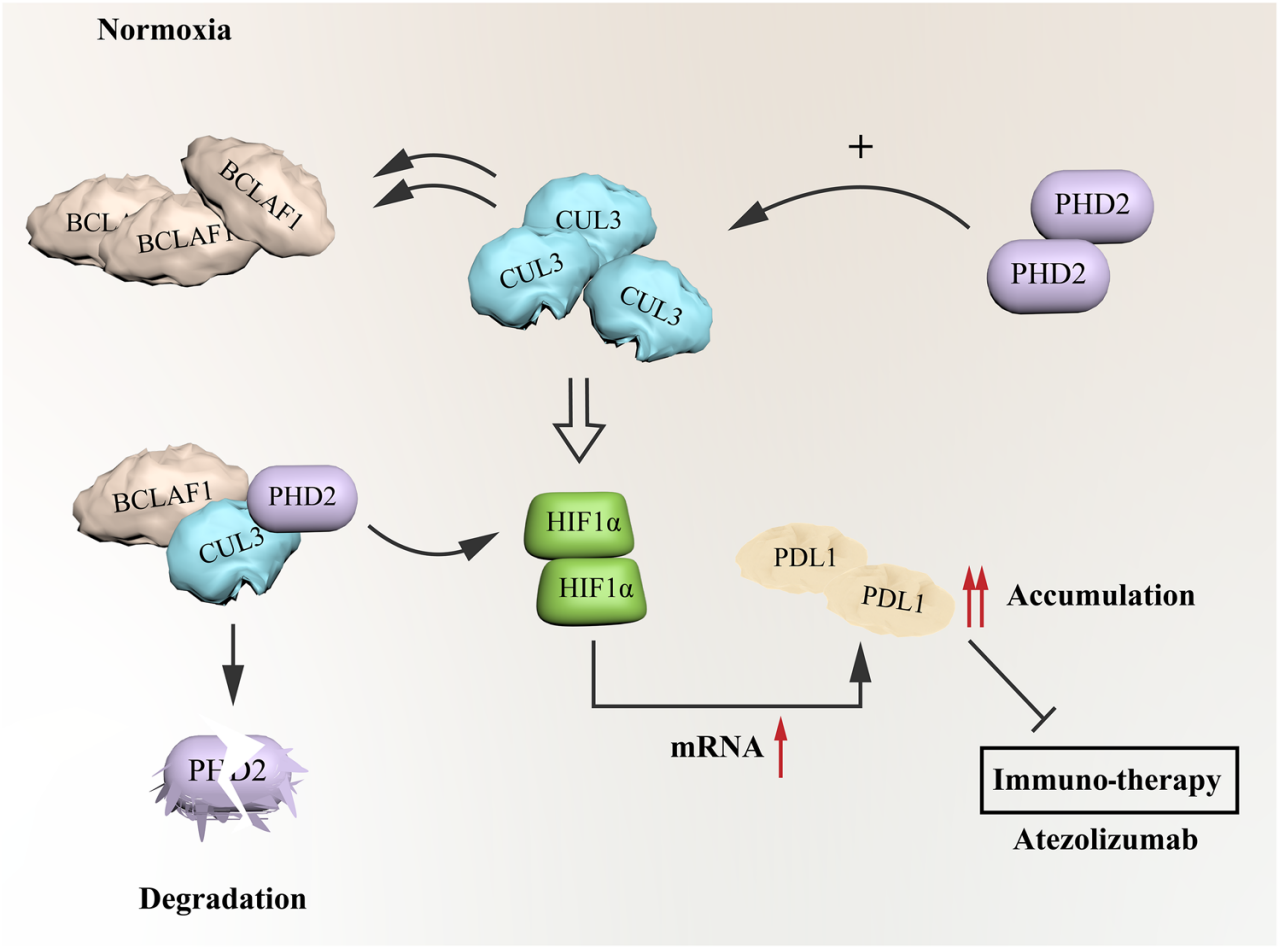
The pattern of BCLAF1-CUL3 complex accumulating HIF-1*α* via degradation of PHD2 and increasing transcription of PD-L1

An expanding body of empirical data has established the pivotal role of BCLAF1 as a potent instigator of tumorigenesis [31–35]. Furthermore, studies have established the association between BCLAF1 and HIF-1*α*, revealing that BCLAF1 is a transcriptional regulator of HIF-1*α* and thereby involved in HIF-1*α* protein accumulation [18]. Our findings reveal the consequential interplay between BCLAF1 and CUL3, providing compelling evidence that the BCLAF1-CUL3 interaction modulates the ubiquitination and subsequent degradation of PHD2 via the proteasomal pathway, ultimately culminating in the accumulation of HIF-1*α* protein under normoxic conditions (Fig. 7).

The expression level of HIF-1*α* is predominantly controlled by VHL, through leveraging oxygen-dependent proline hydroxylation. Typically, in normoxic milieus, VHL-mediated degradation of HIF-1*α* occurs promptly and with remarkable precision through the ubiquitin proteasome system [36]. Of the various PHD isoforms, prolyl hydroxylase domain-containing protein 2 (PHD2) exerts the most discernible influence on the ubiquitination and subsequent degradation of HIF-1*α* [37, 38]. Multiple signaling cascades work together to regulate the stability of HIF-1*α*. For instance, phosphatidylinositol-4,5-bisphosphate-3-kinase (PI3K) orchestrates the activity of kinase B (Akt) and its downstream effector, mammalian target of rapamycin (mTOR), to govern the translational machinery governing HIF-1*α* [39]. Moreover, it has been shown that the mouse double minute 2 (Mdm2) homolog governs the degradation of HIF-1*α* through direct interaction with HIF-1*α*, even under normoxic conditions [40]. Our experimental results reveal that the promotion of PHD2 ubiquitination and subsequent degradation effectively engenders an increase in HIF-1*α* levels.

In contrast to earlier studies about the role of BCLAF1 in enhancing HIF-1*α* transcription under normoxic conditions, our findings underscore the pivotal significance of the CUL3-PHD2 axis in governing HIF-1*α* stability. Notably, upregulating BCLAF1 during normoxia does not result in a significant elevation in HIF-1*α* mRNA levels. We posit that in the absence of VHL or PHD elimination or silencing, the translation of HIF-1*α* mRNAs into functional proteins relies on their stabilization, which then effectively modulates downstream targets. While our data deviate from those of prior studies, it is conceivable that the instability of the protein in normoxic environments may contribute to these discrepancies.

The Cullin-RING family represents the largest assemblage of E3 ligases, and the Cul3 E3 ligase complex, as a constituent member, actively engages in the ubiquitination of diverse proteins [41]. Notably, Kelch ECH-associated protein 1 (Keap1) and the speckle-type POZ protein (SPOP) are recognized as cancer-associated adaptors of CUL3, exerting pivotal roles in tumor progression. Extensive investigations have unequivocally established that Kelch ECH-associated protein 1 (KEAP1) binds to CUL3 through its interaction with the BTB domain located at the N-terminal region of CUL3. Additionally, KEAP1 interfaces with the Kelch domain of nuclear factor erythroid 2-related factor 2 (NRF2), thereby effectively bridging NRF2 and CUL3—the scaffold protein of the E3 ligase RBX1—to regulate NRF2 ubiquitination and proteasomal degradation [42]. In turn, NRF2 promotes tumor cell survival through its antioxidant effect and transcriptional upregulation of Bcl-2 and Bcl-xL [42, 43]. Hence, the CUL3-KEAP1 axis exerts a significant impact on cell cycle regulation, apoptosis, and autophagy [44, 45]. Furthermore, SPOP has recently emerged as a CUL3 adaptor, primarily influencing cytoplasmic signaling and genomic modifications [46]. Its tumor suppressive functions have been confirmed in various malignancies, including prostate cancer, gynecological malignancies (e.g., ovarian, mammary, and endometrial carcinoma), digestive malignancies, and non-small cell lung cancer [47]. More recently, the CUL3-KEAP1 complex has been found to mediate PHD2 polyubiquitination and degradation [36]. In this study, we provide the first evidence of the interaction between BCLAF1 and CUL3, and we further elucidate the critical role of the BCLAF1-CUL3 complex in promoting the ubiquitination and degradation of PHD2 under normoxic conditions. We postulate that BCLAF1 might perform this modulation via specific domains or through epigenetic modifications. Further investigations are warranted to determine the related modification levels, which may involve glycosylation, phosphorylation, and acetylation, along with structural protein analyses. These experiments are likely to be a formidable undertaking, demanding time and resources that exceed our present capacity. Notably, we are the first to explore BCLAF1 involvement in a complex that influences PHD2 degradation. While the current findings do not definitively identify the precise role of BCLAF1 as a member of the ubiquitin-transferase family, they preliminarily confirm the ability of BCLAF1 to bind CUL3 and initiate the PHD2 degradation process, thus underscoring its significance in influencing HIF-1*α* accumulation under normoxic conditions.

However, consistent with prevailing knowledge, the activities of HIF-1*α* influence the transcriptional and expressive aspects of VEGF*α* [47, 48]. Our findings, although not explicitly depicted, indicate that hypoxic conditions foster elevated HIF-1*α* levels, which are not accompanied by a significant increase in VEGF*α* levels. Conversely, the increase in BCLAF1 levels, which promotes HIF-1*α* stabilization under normoxic conditions, does not coincide with an observable increase in VEGF*α*. While VEGF*α* orchestrates angiogenesis, it is just one of many interacting signaling pathways. Histologically, no marked discrepancy in VEGF*α* level was discerned between the two cohorts. Presently, explaining this phenomenon challenges us, but it is potentially caused by the influence and/or expression of other sundry factors. The normoxia-driven increase in HIF-1*α* stability, it seems, is circumscribed, and contrasts with the expected response during hypoxia. We proffer a tentative explanation: the HIF-1*α* protein that accrues during normoxia might be endowed with partial functionalities, or perhaps be functionally restrained by other pathways activated through BCLAF1 overexpression, thereby having no significant effect upon VEGF*α* expression.

HIF-1*α* protein is profoundly increased under normoxic conditions through the overexpression of BCLAF1, highlighting its active and functional state. This elevated expression not only facilitates HCC proliferation and angiogenesis but also orchestrates the transcriptional upregulation of PD-L1. These findings elucidate why heightened PD-L1 levels are observed in patients treated with T + A regimens. Remarkably, the significant upregulation of BCLAF1 activates HIF-1*α*, indirectly promoting PD-L1 transcription and counteracting the therapeutic effects of atezolizumab. Intriguingly, HIF-1*α* function appears to be limited, as we did not observe the upregulation of VEGF*α* following HIF-1*α* accumulation in our normoxic cells. Although VEGF*α* is a classical downstream factor of HIF-1*α* [47, 48], the transcriptional activation of VEGF*α* necessitates higher and more stable levels of HIF-1*α*. This explains why hypoxic culture significantly upregulates VEGF*α* while BCLAF1 overexpression alone does not, thereby elucidating why significant differences in VEGF*α* levels were not observed in tissue samples.

Evaluation of the five pertinent T + A samples through IHC staining revealed that increased BCLAF1, HIF-1*α*, and PD-L1 levels impeded the efficacy of T + A treatment, while treatment with the VEGF*α* antibody demonstrated effectiveness. In the future, leveraging tissue biopsy technology could expedite the identification of potential PD-L1-resistant patients, and the integration of BCLAF1 inhibitors may be used to augment the efficacy of immunotherapy.

In conclusion, based on clinical samples resistant to atezolizumab + bevacizumab, the most potent currently available combination regimen of targeted immunotherapy, this study identified BCLAF1 as a potential regulator of resistance to PD-L1 monoclonal antibody therapy. Subsequently, we treated mouse models with PD-L1 monoclonal antibody therapy in conjunction with BCLAF1 inhibitors to explore the prospective therapeutic effects of anti-BCLAF1 in hepatocellular carcinoma.

### Supplementary Information

Below is the link to the electronic supplementary material.

Fig. 1 A Overall survival of HCC patients curve based on BCLAF1 mRNA expression (TIMER database). B BCLAF1 protein expression in HCC cell lines. C Western blotting tests showed IP antibody of BCLAF1 or CUL3 could connect the protein themself. Statistical significance was denoted as *p < 0.05 (TIF 5260 KB)

Fig. 2 Double immunofluorescence staining of Huh7 and Hep3B cells (TIF 4740 KB)

Fig. 3 Western blotting of CUL3-KEAP1 axis. A Upregulated BCLAF1 had no effect on expression of CUL3-KEAP1. B Inhibition of CUL3 or KEAP1 could decrease expression of PHD2 (TIF 5057 KB)

## Data Availability

The datasets used and/or analyzed during the present study are available from the corresponding author upon reasonable request.
