# Supplementary figures and images for: BCLAF1-induced HIF-1α accumulation under normoxia enhances PD-L1 treatment resistances via BCLAF1-CUL3 complex
Source: Cancer Immunol Immunother. 2023 Oct 31;72(12):4279–92. doi: 10.1007/s00262-023-03563-8 (PMC10700218; doi:10.1007/s00262-023-03563-8)

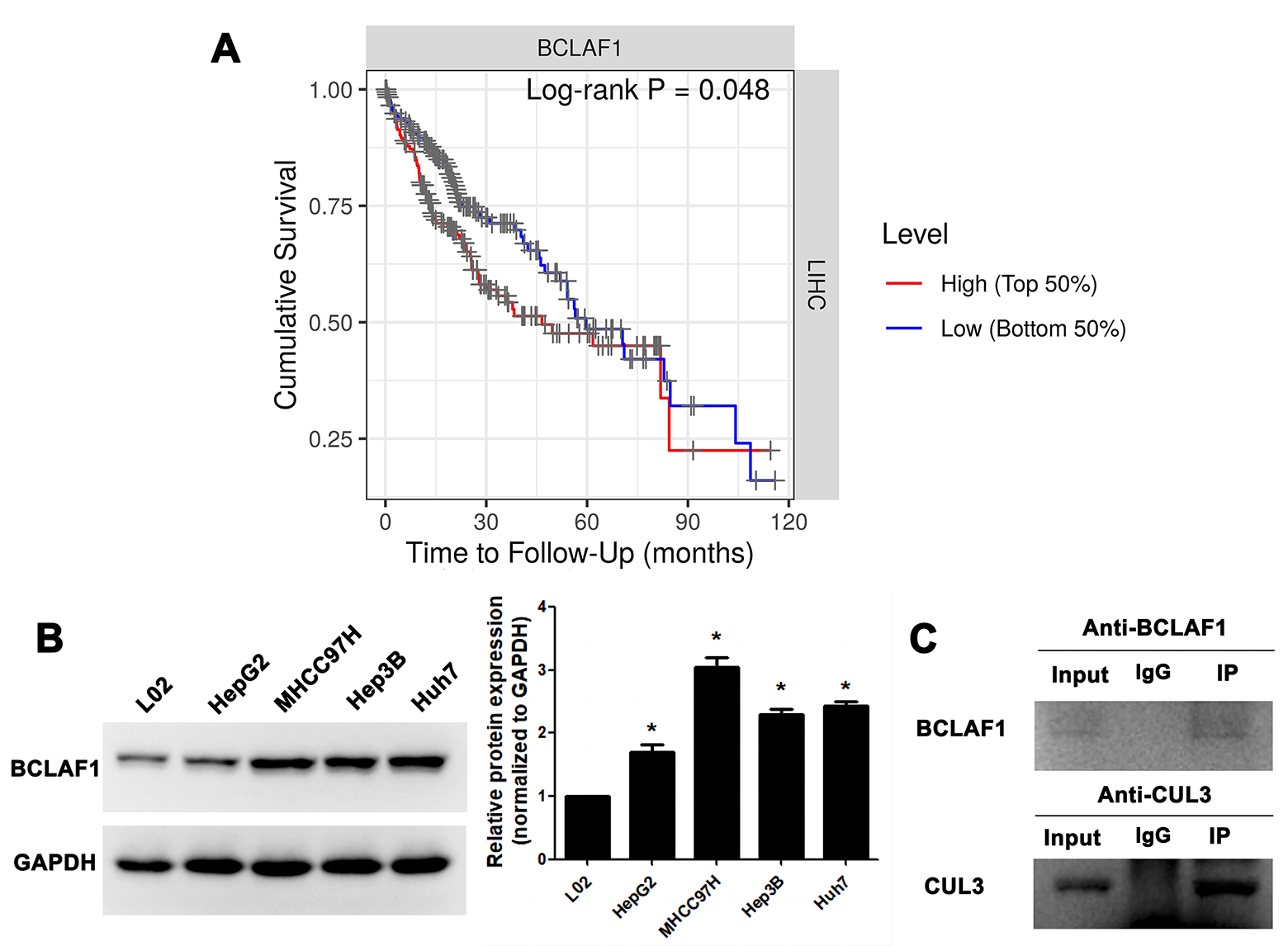

Supplement: Supplementary file 1 — Fig. 1 A Overall survival of HCC patients curve based on BCLAF1 mRNA expression (TIMER database). B BCLAF1 protein expression in HCC cell lines. C Western blotting tests showed IP antibody of BCLAF1 or CUL3 could connect the protein themself. Statistical significance was denoted as *p < 0.05 (TIF 5260 KB) [file 262_2023_3563_MOESM1_ESM.tif]

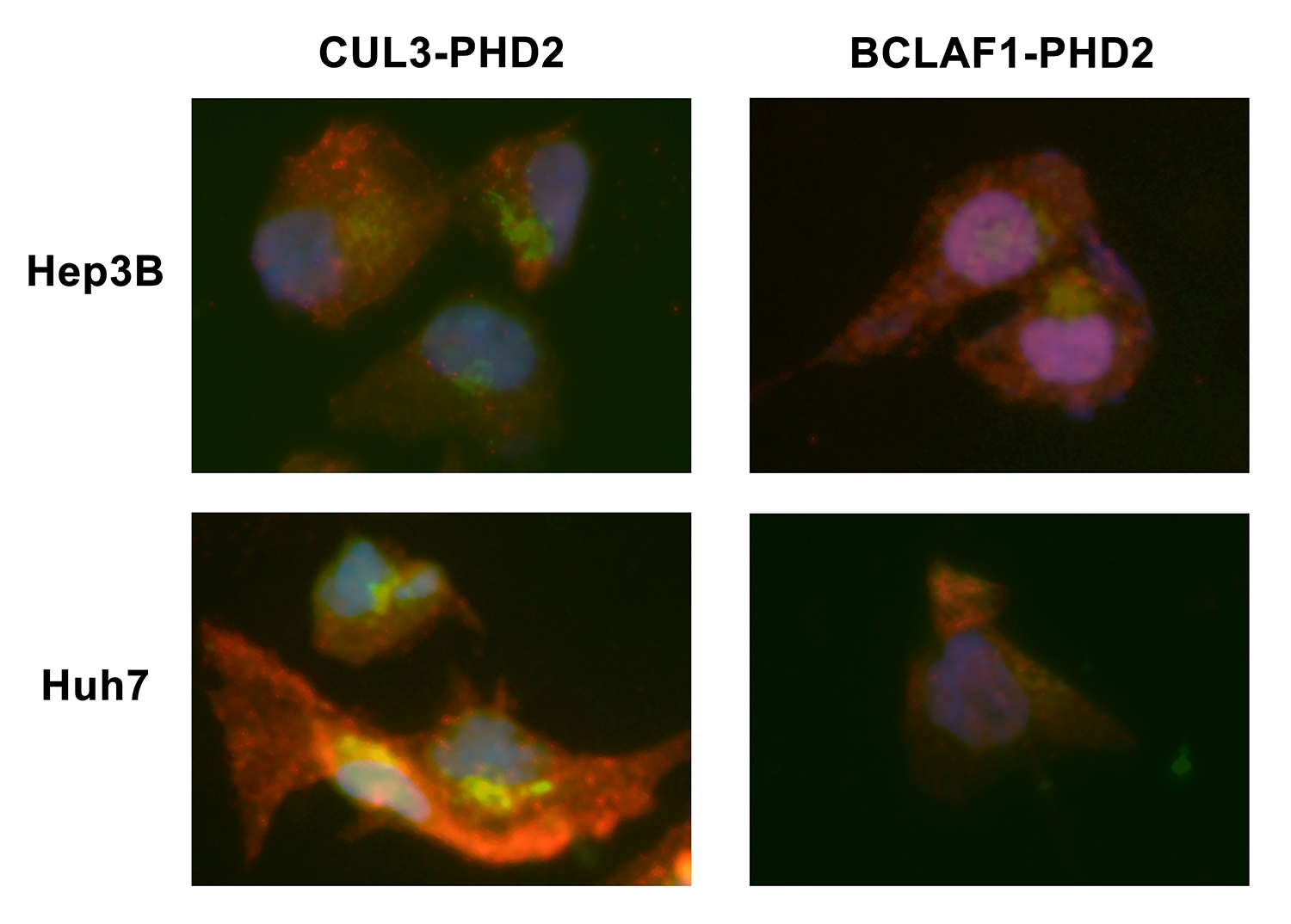

Supplement: Supplementary file 2 — Fig. 2 Double immunofluorescence staining of Huh7 and Hep3B cells (TIF 4740 KB) [file 262_2023_3563_MOESM2_ESM.tif]

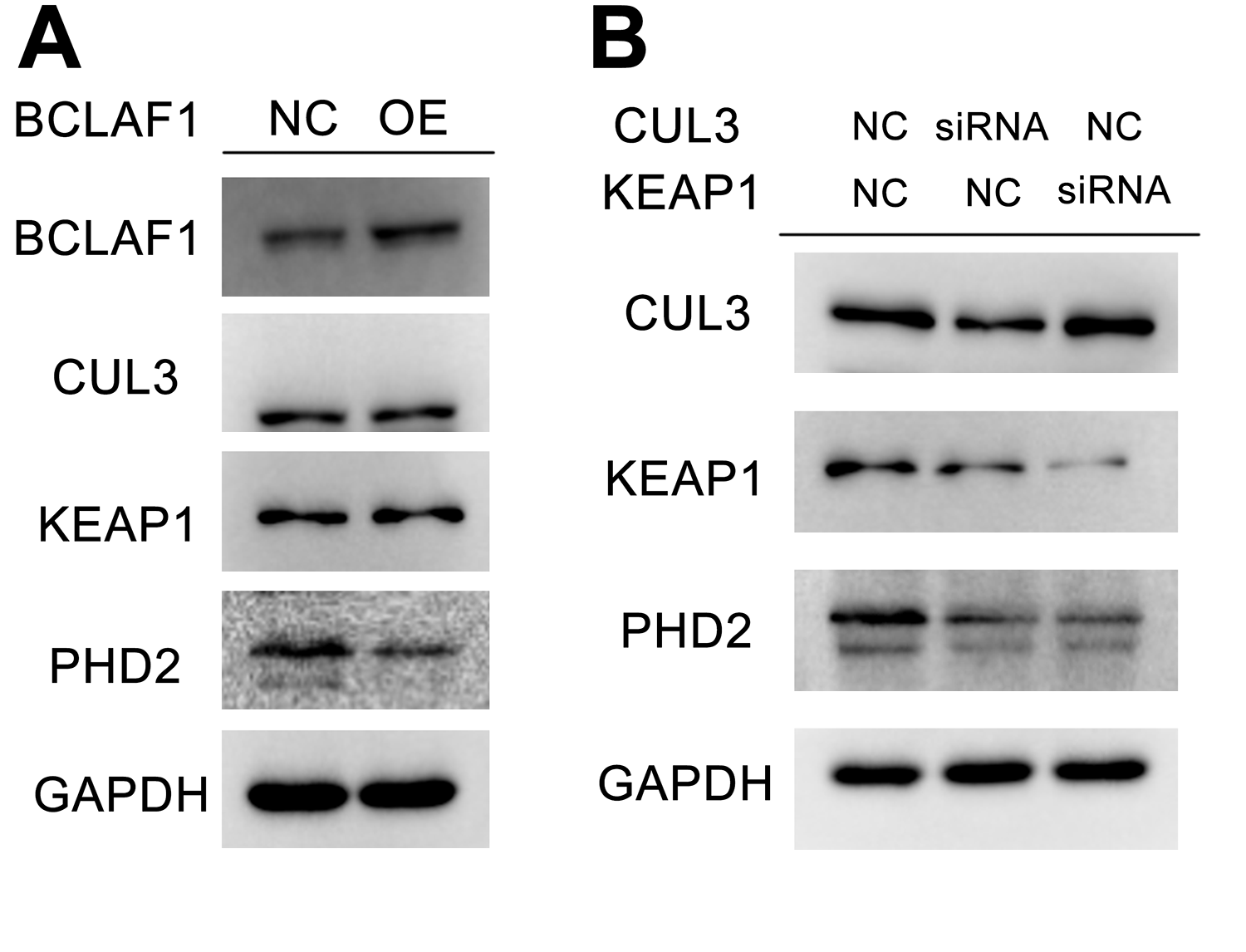

Supplement: Supplementary file 3 — Fig. 3 Western blotting of CUL3-KEAP1 axis. A Upregulated BCLAF1 had no effect on expression of CUL3-KEAP1. B Inhibition of CUL3 or KEAP1 could decrease expression of PHD2 (TIF 5057 KB) [file 262_2023_3563_MOESM3_ESM.tif]
